# The complete mitochondrial genome of *Geochelone sulcata*

**DOI:** 10.1080/23802359.2017.1357437

**Published:** 2017-07-27

**Authors:** Qiong Shi, Jue Wang, Jianjun Liu, Hui Jiang, Liuwang Nie

**Affiliations:** Life Science College, Provincial Key Lab of the Conservation and Exploitation Research of Biological Resources in Anhui, Anhui Normal University, Wuhu, China

**Keywords:** *Geochelone sulcata*, mitochondrial genome, evolutionary relationships

## Abstract

The complete mitochondrial genome of *Geochelone sulcata* was determined using PCR, Long-PCR with length of 16,692 bp. The genome organization, gene order, and base composition was similar to typical vertebrate. Gene content included 13 protein-coding genes, 22 tRNA genes, two rRNA genes, and one control region. Otherwise, the lack of C, as same as in the other species of Testudinidae, was detected in arms of *tRNA^Lys^* gene in *G. sulcata*. In addition, an extra nucleotide A was discovered in *ND3* gene in *G. sulcata*. The complete mitogenome of *G. sulcata* provides the basic data to research molecular systematics of Testudinidae.

*Geochelone sulcata* is the largest tortoise of the African mainland, distributed in Ethiopia (ET), Sudan (SD), Senegal (SN), Mali (ML), and other countries (Wiesner and Iben 2003). Because of habitat destruction, overhunting and environmental pollution, the wild populations have decreased dramatically (Heinrich and Heinrich 2016). Consequently, International Union for Conservation of Nature (IUCN) has included it in the Red List of Endangered species (Version 3.1, 2012). At present, only partial mitochondrial genome of *G. sulcata* has been published on NCBI (Lopez-Oceja et al. 2016), the complete mitochondrial genome sequence hasn’t been reported.

*G. sulcata* was collected from Nanjing Zoo, Jiangsu province of China (CN) (32.09°N, 118.81°E) and stored in the herbarium of Anhui Normal University. Total genomic DNA (Code No.26080216) was extracted from the tip tail tissues (Length: 3–5mm) by phenol–chloroform method (Zhou et al. 2015). The mitochondrial sequence was amplified by PCR and Long-PCR. The sequence assembly of the mitochondrial genome by DNAStar version 5.0 software (Shi et al. 2003). Using SEQUIN9.0 software to find the protein coding gene (Kan et al. 2010). The tRNA gene was localized with tRNAscan-SE1.21 software (http://lowelab.ucsc.edu/tRNAscan-SE). The rRNA gene and control region are determined by comparison with other turtles (Lan et al. 2015; Li et al. 2017). The complete sequence of *G. sulcata* was submitted to GenBank for accession number KJ489404.

The complete mitochondrial genome of *G. sulcata* has a circular genome of 16,692 bp and consists of 13 protein-coding genes, two rRNA genes, 22tRNA genes, and one control region. The contents of T, C, A, and G are 24.4%, 28.2%, 35.0%, and 12.5%, respectively. Among them, G is the least, the results show that the anti-G bias of mtDNA. All the protein-coding genes encoded on the heavy strand, except ND6 encoded on the light strand. The lengths of 12S rRNA and 16S rRNA are 973 bp and 1607 bp, respectively. The length of D-loop is 945 bp, ranging from 15,583 to 16,527 bp. In general, there is an additional C in arms of *tRNA^Lys^* gene of non-testudinidae species that forms a small ring, whereas the mitochondrial gene of *G. sulcata*, there is no additional C. This phenomenon has been reported in other species of Testudinidae (Parham et al. 2006), and this feature can be used as an identification feature of the mitochondrial genome of Testudinidae. In addition, an extra nucleotide A was discovered in *ND3* gene in *G. sulcata*, which allows the codon of the encoded protein to terminate.

Based on using mt thirteen protein-coding genes of *G. sulcata* and the other 17 species of Testudinidae, using *C. reevesii* and *C. flavomarginata* as outgroup, phylogenetic relationships were inferred with maximum likelihood (ML), Maximum parsimony (MP), and Neighbor-Joining (NJ). Phylogenetic trees clearly indicated a sister relationship between *G. sulcata* and *G. elegans*. Manouria at the end of the cladogram. From the phylogenetic tree, we found that Indotestudo nested inside of Testudo (Figure 1) (van der Kuyl et al. 2002; Le et al. 2006; Luján et al. 2014). The evolutionary relationships of these species are consistent with the previously reported results, but the phylogenetic positions among them have never been well resolved.

**Figure 1.**
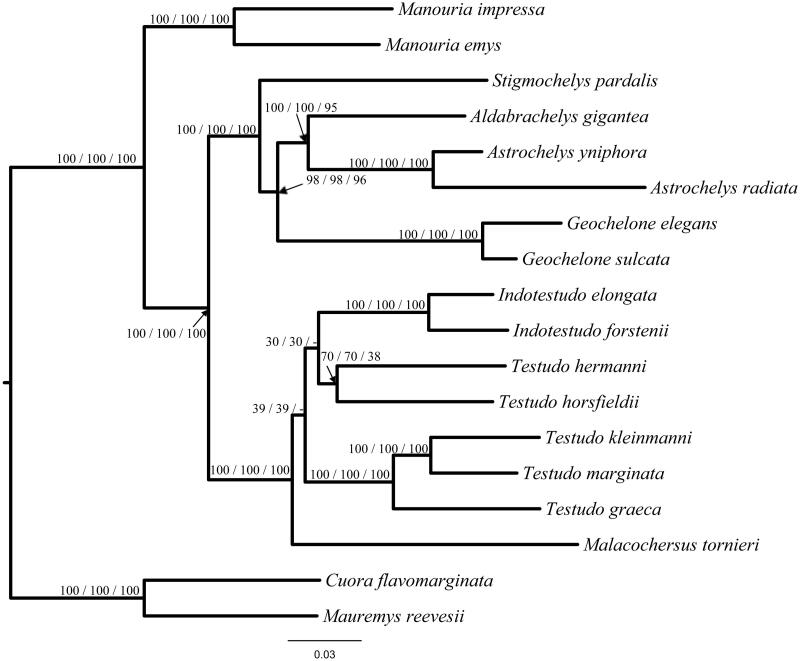
Phylogenetic trees for *G. sulcata* and the other 17 species reconstructed based on mt thirteen protein-coding genes, using Chinemys reevesii and Cuora flavomarginata as outgroup. Number above each node indicates the bootstrap support values recovered from ML, NJ, MP analyses, respectively. All 18 species’s accession numbers are listed as below: *Indotestudo elongate* NC_007695*, Indotestudo forstenii* NC_007696*, Testudo hermanni* DQ080046*, Testudo horsfieldii* NC_007697*, Testudo graeca* NC_007692*, Testudo kleinmanni* NC_007699*, Testudo marginata* NC_007698*, Malacochersus tornieri* NC_007700*, Stigmochelys pardalis* DQ080041*, Aldabrachelys gigantean* NC_028438*, Astrochelys radiate* KJ489403*, Astrochelys yniphora* JX317746, *G. sulcata* KJ489404*, Geochelone elegans* KJ489405*, Manouria emys* NC_007693*, Manouria impressa* NC_011815*, C*. *flavomarginata* EU708434, *M. reevesii* AY676201.
